# Study of Three-Phase Flow Field Characteristics in a Multi-Stage Friction–Shear Cavitating Waterjet for Flake Graphite Liberation

**DOI:** 10.3390/ma19142961

**Published:** 2026-07-09

**Authors:** Xing Dong, Yun Jiang, Deqiang Peng, Jiaxing Li, Dongsheng Li

**Affiliations:** 1 School of Mechanical and Electrical Engineering, Guangdong University of Science and Technology, Dongguan 523083, China; 2 School of Mechanical Engineering, Heilongjiang University of Science and Technology, Harbin 150022, China; 15754502213@163.com (J.L.); 13658204671@163.com (D.L.); 3 School of Safety Engineering, Heilongjiang University of Science and Technology, Harbin 150022, China; jiangyun011010@163.com; 4 School of Mining Engineering, Heilongjiang University of Science and Technology, Harbin 150022, China; barrypdf01@163.com

**Keywords:** multi-stage friction–shear cavitating waterjet nozzle, numerical simulation, vapor volume fraction, flake graphite, fixed carbon content

## Abstract

Flake graphite is a natural non-metallic material with excellent electrical and thermal conductivity and good lubricity. This study proposed a multi-stage friction–shear cavitating waterjet method to enhance the liberation of flake graphite from gangue minerals. A corresponding nozzle was designed and fabricated by integrating liquid–solid two-phase transport, grinding kinetics, profile-based design, and similarity design. Fluent simulations were conducted with the Eulerian multiphase model to analyze the water–vapor–flake graphite three-phase flow field at different inlet pressures, focusing on vapor volume fraction, water phase flow, and flake graphite particle phase behavior. A distinct cavitation region appeared in the outlet diverging section, mainly near the wall. At inlet pressures of 25 MPa and above, the maximum vapor volume fraction was maintained above 99%, suggesting strong cavitation-inducing capability. Jet liberation experiments showed that the fixed carbon content increased from 49.11% in the feed sample to 78.77% in the waterjet-treated flotation concentrate, while D90 decreased from 121.36 to 103.33 μm and the average particle size decreased from 62.78 to 55.02 μm. These results indicate that multi-stage friction–shear cavitating waterjet treatment facilitates the liberation of flake graphite from gangue minerals, thereby improving the fixed carbon content of the flake graphite concentrate.

## 1. Introduction

Cavitation is a complex phase transition process in liquids that is generally triggered when the local pressure falls to a level close to or lower than the saturated vapor pressure. It essentially involves the generation, development, and collapse of vapor bubbles [1,2,3]. Under the combined effects of pressure gradients, wall constraints, and unsteady flow, bubble collapse can induce microjets, shock waves, and local high-pressure pulsations, thereby causing noise, vibration, and material erosion [3,4,5,6]. In early studies, cavitation was mostly regarded as an undesirable phenomenon in hydraulic machinery. However, its transient energy release characteristics also provide a new approach for enhancing jet action [1,2]. Cavitating waterjets utilize this pressure reduction mechanism to generate low-pressure zones within high-speed jets or along shear layers through specific nozzle geometries, thereby enabling the sustained formation and evolution of cavitation bubbles in the flow field. When these bubbles collapse near a target surface or wall region, local impact energy can be released [2,7]. Therefore, compared with conventional waterjets, cavitating waterjets not only retain the impact and shear effects of ordinary jets but also introduce additional impact effects generated by bubble collapse, which is beneficial for improving jet energy utilization efficiency. Owing to these advantages, cavitating waterjets have been widely applied in rock breaking [8,9,10], cleaning and decontamination [11,12,13], and cavitation peening [14,15].

At present, considerable attention has been paid to the application of cavitating waterjets in mineral comminution. Sen [16] used a waterjet-driven cavitation cell to grind magnetite and found that inlet pressure, standoff distance, treatment time, and feed particle size all affected the particle size of the products. Galecki et al. [17] carried out coal particle comminution tests using a cavitation cell and indicated that back pressure and the number of circulation passes affected fine particle generation. Galecki et al. [18] further examined the roles of feed particle size, ash content, and inlet pressure in coal particle comminution. Guo et al. [19] used a cavitation abrasive waterjet to comminute mica and pointed out that shear and cavitation played key roles in the comminution of layered mica. Li et al. [20] used a high-pressure waterjet cavitation cell coupling device to comminute coal particles and found that higher operating pressure was favorable for obtaining finer products.

Overall, existing studies on cavitating waterjets in mineral processing have mainly aimed to improve particle breakage efficiency and achieve particle size reduction, with a focus on particle refinement and comminution behavior. However, for typical layered minerals such as flake graphite, their application value highly depends on the integrity of the flake structure and the yield of large flakes. Therefore, flake graphite liberation should not simply pursue particle refinement; rather, it should emphasize the liberation between graphite and gangue minerals under relatively mild action conditions. Nevertheless, little attention has been paid to how cavitation, shear, and impact effects can be used to promote interfacial separation between graphite and gangue minerals, and specialized cavitating waterjet nozzles designed for flake graphite liberation remain insufficiently studied.

As the key flow channel component of a cavitating waterjet system, the nozzle has a decisive influence on cavitating waterjet performance. In recent years, CFD-based simulations have been widely adopted and have proven effective for exploring cavitating waterjet mechanisms and optimizing nozzle design. A series of achievements has been made in the numerical simulation of cavitating waterjet mechanisms. Zhang et al. [21] analyzed the transient evolution of an artificially submerged cavitating waterjet using LES in conjunction with the Schnerr–Sauer cavitation model. Wang et al. [22] examined the influence of curved-surface confinement on the impact behavior of self-excited oscillating cavitating jets by integrating high-speed visualization and computational modeling. Du et al. [23] applied SBES to submerged cavitating jets and demonstrated its ability to resolve fine-scale turbulent structures and cavitation cloud shedding in the downstream region. Onishi et al. [24] developed a compressible mixture-flow cavitation model incorporating phase change effects to reproduce cloud shedding periodicity and associated pressure pulsations in high-speed submerged waterjets. Liu et al. [25] examined three nozzle configurations—cylindrical, organ pipe, and converging–diverging types—and revealed that cavitation cloud deformation and breakup were driven by a re-entrant jet. Xu et al. [26] assessed the performance of the SST *k*–*ω*, LES, and SBES models and reported that LES and SBES captured transient cavitation cloud evolution more effectively. Dong et al. [27] investigated a submerged angular cavitation nozzle using the Mixture and RNG *k*–*ε* models and obtained a structural parameter combination favorable for enhancing cavitation.

Using CFD techniques, researchers have also carried out extensive studies on flow field analysis during nozzle design. Guo et al. [28] developed a co-flow cavitating waterjet nozzle and observed a periodic cavitation cloud evolution characterized by bubble inception, cloud growth, cloud shedding, and collapse. Fang et al. [29] used a three-dimensional LES method to study an organ pipe nozzle and pointed out that inlet pressure affected the dominant jet frequency, whereas the divergence angle mainly affected the oscillation amplitude. Yue et al. [30] examined cavitation nozzles with varied divergence angles for strengthening 7075 aluminum alloy, finding that the divergence angle changed the outlet velocity and dynamic pressure distribution. Guo et al. [31] combined CFD with a Kriging differential evolution algorithm to optimize the diffusion section of an angular Helmholtz nozzle. Zheng [32] constructed a double-chamber self-resonating cavitation nozzle and found that a chamber length ratio of 0.96 and a chamber diameter ratio of 3 were favorable for enhancing cavitation. Dong et al. [33] proposed an arc-shaped chamber nozzle and found that using a 2 mm arc radius markedly enlarged the cavitation region and increased the turbulent kinetic energy. Huang et al. [34] optimized a composite cavitation nozzle by coupling LES with response surface methodology and identified appropriate values for the connecting channel diameter, Helmholtz chamber diameter, and chamber length. Luo et al. [35] studied an unsubmerged coaxial double-nozzle cavitating waterjet and indicated that the inner nozzle structure and incident pressure affected impact pressure and cavitation intensity. Chen et al. [36] used a hybrid RANS–LES model to study a converging–diverging nozzle and found that a divergence angle of 60° provided better cavitation performance. Su et al. [37] analyzed waterjet nozzle cavitation using the Realizable *k*–*ε* model and pointed out that inlet pressure and the throat length-to-diameter ratio influenced the vapor volume fraction distribution. He et al. [38] applied the VOF–LES method to a converging–diverging nozzle and found that enlarging the throat diameter reduced energy consumption but weakened cavitation performance. Gao et al. [39] optimized an angular cavitation nozzle for hydraulic fracturing using the SST *k*–*ω* model and orthogonal experiments and obtained a favorable structural combination.

In general, existing studies have systematically revealed the effects of nozzle structural parameters on water–vapor two-phase cavitating flow fields and have provided an important basis for cavitating waterjet nozzle design. However, current cavitating waterjet studies mainly focus on rock breaking, cleaning and decontamination, cavitation peening, and general mineral comminution. Nozzle structures are usually designed to improve impact performance, enhance cavitation intensity, or promote particle refinement. For layered minerals such as flake graphite, the liberation process differs from the breakage of ordinary brittle particles, and it places different requirements on the coupling relationship among friction–shear action, particle transport characteristics, and cavitation effects inside the nozzle. At present, studies on cavitating waterjet nozzle structures and flow field characteristics designed specifically for flake graphite liberation remain limited, and the influence mechanism of multi-stage friction–shear structures on cavitation development and flake graphite particle motion behavior is still unclear.

Based on these issues, this study proposes a multi-stage friction–shear cavitating waterjet nozzle for the liberation of flake graphite from gangue minerals. The design concept of this nozzle is to make the flake graphite slurry repeatedly undergo acceleration, near-wall shear, local pressure reduction, and cavitation development in the flow channel through multi-stage contraction, friction, and expansion structures. This process can enhance the friction–shear action between particles and the channel wall as well as among particles, while providing favorable conditions for local impact effects related to bubble collapse. Unlike conventional cavitating waterjet devices that mainly aim at particle breakage and particle size reduction, the nozzle structure proposed in this study focuses more on promoting the liberation of flake graphite from gangue minerals. To achieve this design objective, the structure of the multi-stage friction–shear cavitating waterjet nozzle was first designed based on liquid–solid two-phase flow transport, grinding kinetics, profiling design, and similarity design methods. Subsequently, an Eulerian multiphase model was used to numerically simulate the internal and external water–vapor–flake graphite three-phase flow field of the nozzle at different inlet pressures, and the pressure-dependent variations in vapor volume fraction, water phase axial velocity, and flake graphite particle phase axial velocity were examined. Finally, cavitating waterjet liberation and flotation experiments on flake graphite were conducted, and particle size distribution and fixed carbon content were used as evaluation indicators to evaluate the actual liberation effect of the proposed nozzle on flake graphite.

Compared with existing cavitating waterjet studies that mainly focus on rock breaking, cleaning and decontamination, cavitation peening, and general mineral comminution, the novelty of this study mainly lies in the development of a nozzle structure tailored to the liberation requirements of the layered structure of flake graphite, which integrates multi-stage contraction acceleration, near-wall friction–shear action, and cavitation induction in the outlet diverging section. Furthermore, this study combines water–vapor–flake graphite three-phase flow field analysis with experimental evaluation of actual liberation. Numerical simulations were used to reveal the characteristics of the internal water–vapor–flake graphite three-phase flow field in the nozzle, while liberation experiments were conducted to evaluate the feasibility of the multi-stage friction–shear cavitating waterjet nozzle for promoting flake graphite liberation. The results verified the superiority of the proposed nozzle and provided technical support for improving the liberation degree of flake graphite and practical production.

## 2. Materials and Methods

### 2.1. Simulation Parameters

Computational fluid dynamics (CFD) was employed in this study to perform the numerical simulations. Previous studies have demonstrated the suitability of ANSYS Fluent for resolving complex internal flows in nozzles, cavitating waterjets, and multiphase flow problems. By coupling turbulence, multiphase-flow, and cavitation models, ANSYS Fluent can be used to obtain key flow field parameters, such as velocity, pressure, and phase volume fraction. Considering that the multi-stage friction–shear cavitating waterjet nozzle investigated in this study involves a multi-stage series structure, local sudden expansion and contraction, strong turbulence, cavitation-induced phase change, and water–vapor–flake graphite three-phase coupled flow, ANSYS Fluent 2024 R2 was selected as the numerical simulation platform.

In the numerical simulations, liquid water served as the continuous carrier phase in the three-phase flow and was therefore defined as the primary phase. Its density and dynamic viscosity were set to 1000 kg/m^3^ and 1 × 10^−3^ Pa·s, respectively. Since the cavitation bubbles generated by the cavitating jet are mainly composed of water vapor, water vapor was defined as the gaseous secondary phase, with a density and dynamic viscosity of 0.02558 kg/m^3^ and 1 × 10^−5^ Pa·s, respectively. The flake graphite particle phase was defined as the solid secondary phase, with a volume fraction of 15% and a density of 2100 kg/m^3^. Based on the measured particle size distribution of the feed sample, 0.062 mm was used as the representative particle diameter in the Eulerian model. In the numerical model, the flake graphite particle phase was treated as a pseudo-fluid particulate phase under a continuum assumption to describe its macroscopic transport behavior in the slurry. The main physical parameters of each phase in the Eulerian multiphase model are summarized in Table 1.

The inlet pressure was used as the primary analysis variable, with five pressure levels set at 5, 15, 25, 35, and 45 MPa. The pressure-dependent characteristics of the water–vapor–flake graphite three-phase flow field were then examined by varying the inlet pressure of the multi-stage friction–shear cavitating waterjet nozzle.

### 2.2. Multi-Stage Friction–Shear Cavitating Waterjet Nozzle

#### 2.2.1. Nozzle Configuration

**Figure 1:**
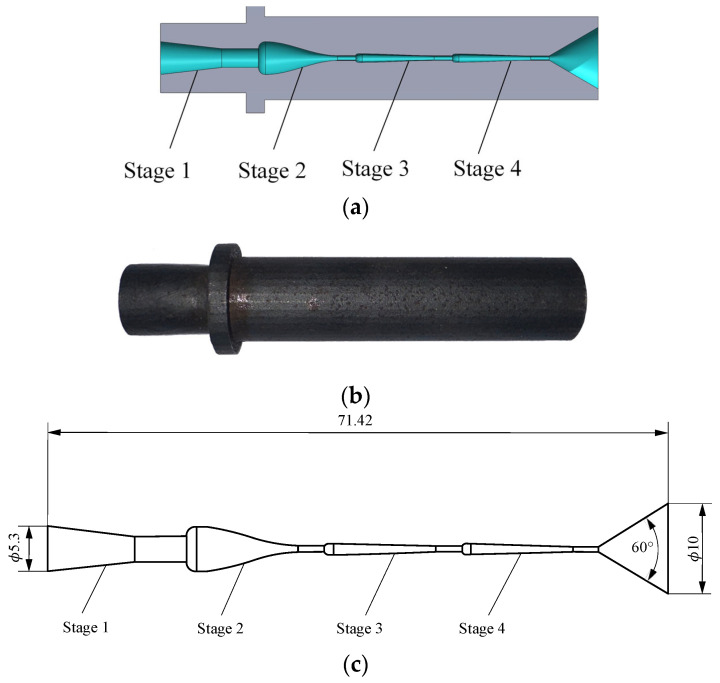
Multi-stage friction–shear cavitating waterjet nozzle. (**a**) Overall structural schematic of the nozzle; (**b**) photograph of the nozzle; (**c**) internal flow channel geometry and key dimensions of the nozzle.

Figure 1 presents the overall structure, photograph, and internal flow channel geometry of the self-developed multi-stage friction–shear cavitating waterjet nozzle. Figure 1a shows the overall structural schematic of the nozzle, Figure 1b shows a photograph of the nozzle, and Figure 1c shows the internal flow channel geometry and key dimensions of the nozzle. As shown in Figure 1a, the nozzle comprises four serially connected nozzle elements with different internal flow channel structures. The first stage is a conical converging nozzle with a cylindrical outlet section, including an inlet conical converging section and an outlet cylindrical section. This stage converts the pressure energy of high-pressure water into kinetic energy, thereby accelerating the liquid water and increasing the axial velocity of flake graphite particles. The second stage is an arc profile streamlined friction nozzle, comprising an inlet arc-shaped diverging section, a middle streamlined converging section, and an outlet friction section. This stage is intended to promote flake graphite liberation through particle wall friction–shear interactions in the outlet friction section. The third stage is a flat, streamlined friction nozzle, including an inlet curved diverging section, a middle streamlined converging section, and an outlet friction section. While reducing turbulence intensity, this stage provides further friction–shear action and thereby assists flake graphite liberation. The fourth stage is a flat, streamlined friction–cavitation nozzle, consisting of an inlet curved diverging section, an upstream middle streamlined converging section, a downstream middle friction section, and an outlet diverging section. On the basis of the third-stage function, the outlet diverging section acts as a diverging section shear cavitation nozzle, which is expected to further enhance flake graphite liberation through microjet impact, water wedge tensile action, and stress wave effects induced by cavitation.

Figure 1c shows the internal flow channel geometry of the self-developed multi-stage friction–shear cavitating waterjet nozzle together with the key geometric dimensions used to characterize the overall nozzle scale, establish the computational domain, and analyze the main flow features. In Figure 1c, the inlet diameter of the first-stage conical converging nozzle with a cylindrical outlet section is 5.3 mm. The friction section orifices of the second-, third-, and fourth-stage nozzles are designed as arc-shaped straight-slot openings with vertical and horizontal symmetry, and their heights all range from 0.6 to 0.7 mm and gradually decrease along the flow direction. For the fourth-stage flat streamlined friction–cavitation nozzle, the diverging section outlet diameter is 10 mm, and the total nozzle length is 71.42 mm.

#### 2.2.2. Nozzle Structural Design

The structural design of the multi-stage friction–shear cavitating waterjet nozzle was based on the principle of fully utilizing the functional role of each nozzle stage.

The first-stage conical converging nozzle with a cylindrical outlet section was designed based on the principles of liquid–solid two-phase hydraulic transport and waterjet nozzle design. The conical converging section of the first-stage nozzle is connected to the high-pressure hose, and the graphite slurry flow in the high-pressure hose-nozzle system can be regarded as a hydraulic transport process of flake graphite particles. As the flake graphite–water slurry enters the conical converging section of the nozzle, the converging structure converts the pressure energy of water into kinetic energy, thereby accelerating the water phase. This process generates a new velocity difference between the flake graphite particles and water. Consequently, the flake graphite particles are further accelerated by water drag and then enter the outlet cylindrical section. Therefore, the converging angle is an important factor governing the energy conversion and transfer of the high-pressure water. According to the waterjet nozzle design method, the conical converging angle of the nozzle was set to 13.5° [27,36], which is beneficial for the formation of a coherent jet region. The outlet cylindrical section provides stable high-speed flow conditions for the jet. Assuming that the flake graphite particles remained suspended, the outlet cylindrical-section diameter d was calculated as follows [28]:

(1) $$d=\sqrt{\frac{4Q}{\pi \mu \sqrt{\frac{2p}{\rho }}}}$$

where *Q* is the nozzle flow rate (m^3^/s); *μ* is the flow coefficient; *p* is the jet pressure (Pa); and *ρ* is the slurry density (kg/m^3^). The length of the outlet cylindrical section was taken as twice its diameter, which helps to form a reasonably concentrated jet region while reducing energy loss.

The friction section orifices of the second-, third-, and fourth-stage nozzles were designed based on the principles of liquid–solid two-phase hydraulic transport, grinding kinetics, profile-based design, and geometric similarity. From the perspective of liquid–solid two-phase hydraulic transport and grinding kinetics, the main friction–shear action on flake graphite particles occurs in the friction sections as the graphite slurry passes through the multi-stage friction–shear cavitating waterjet nozzle. Therefore, the rational design of the cross-sectional profile and dimensions of the internal flow channel in each friction section orifice, together with an enlarged frictional contact area between the internal wall of the friction section and the flake graphite particles, is expected to enhance the friction-assisted liberation performance of the nozzle.

In this study, a profile-based design method was adopted for the friction section orifices. The design procedure consisted of the following four steps.

**Figure 2:**
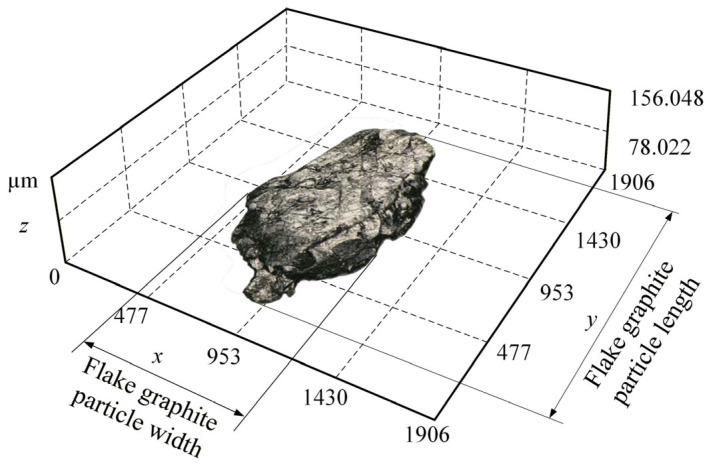
Three-dimensional morphology image of a representative flake graphite particle.

First, the surface morphological characteristics of the flake graphite particles were determined. Referring to the shape parameters used for aggregate particle characterization, the length, width, and thickness were adopted to describe the morphological features of the flake graphite particles. Specifically, flake graphite particles were randomly selected from the prepared samples, and their morphologies were observed using a DSX-1000 digital microscope (Olympus Corporation, Tokyo, Japan), which was used to acquire particle morphology images and measure geometric parameters. The length, width, area, perimeter, and other morphological parameters of each particle were measured and statistically analyzed. Meanwhile, the width-sectional profile curve of each particle was generated by the software, and the particle thickness was obtained to assist in verifying the measurement accuracy and providing basic data for the profile-based design. Figure 2 shows a three-dimensional morphology image of a representative flake graphite particle selected from the above-observed samples, which is used to display the typical flat layered morphological characteristics of the flake graphite particles in this sample. It should be noted that the particle shown in Figure 2 is used only to visually illustrate the typical morphology of flake graphite particles. The morphological parameters used for the profile-based design of the orifice, including length, width, thickness, and profile curves, were obtained from the measurement results of multiple randomly selected flake graphite particles. As shown in Figure 2, the flake graphite particle generally exhibits a flat, flake-like layered structure, with obvious bedding along the lamellar direction and close interlayer connection. In addition, irregular small pits and protrusions are randomly distributed on the graphite surface.

Second, the width-direction cross-sectional profile curves of the flake graphite particles were fitted. Based on the hydraulic transport theory for liquid–solid two-phase flow, the flake graphite slurry exhibits a highly turbulent flow state in the friction sections of the multi-stage friction–shear cavitating waterjet nozzle. When the particle long axis is parallel to the nozzle axis, frictional contact between the broad surfaces of the high-speed flat flake graphite particles and the upper/lower internal walls of the friction section channel remains highly stochastic. Therefore, to better conform to the actual physical situation, the upper/lower and left/right fitted curves of the width-direction particle cross-sectional profile were assumed to be symmetric. Accordingly, a symmetric fitting strategy was adopted during curve fitting. The least-squares circular-arc fitting method was used to fit the upper and left profile curves of the particle cross section along the width direction. The results showed that both the upper and left fitted curves could be approximated as circular arcs with different curvature radii. Based on this result, the width-direction cross-sectional profile of the flake graphite particles was defined as paired upper/lower symmetric circular arcs and paired left/right symmetric circular arcs, with each pair sharing the same curvature radius.

Therefore, the width-direction characteristic dimension of the orifice was determined from the projected envelope of flake graphite particles within the orifice cross-section. The equivalent trajectory band width *b_f_* of the particle in the width direction can then be expressed as follows:

(2) $$b_{f}=l_{1}+l_{2}\sin\beta$$

On this basis, considering that a certain safety clearance should be reserved between the particle and the wall when the particle passes through the orifice, the chord length *b*_1_ of the upper arc of the width-sectional profile of the friction section orifice can be expressed as follows:

(3) $$b_{1}=b_{f}+2d_{f}$$

where *b_f_* is the trajectory band width of the flake graphite particle (mm); *d_f_* is the distance from the upper arc of the flake graphite particle width-sectional profile to the upper wall of the orifice (mm), and *d_f_* = 0.25*b_f_* in this study; *l*_1_ is the length of the flake graphite particle (mm); *l*_2_ is the width of the flake graphite particle (mm); and *β* is the deflection angle of the flake graphite particle, which was taken as 3° in this study.

According to Equations (2) and (3), the chord length *b*_1_ for the upper arc of the friction section orifice width profile can be obtained. The ratio of *b*_1_ to the particle width *l*_2_ was then used as the scaling factor. Based on geometric similarity, the curvature radius of the upper arc of the particle width-sectional profile was multiplied by this scaling factor to obtain the corresponding curvature radius of the upper arc of the orifice width-sectional profile. The curvature radius of the left profile of the orifice width section was determined using the same method. According to the geometric symmetry of the orifice structure with respect to the centerline, the complete profile of the cross-section was then determined. It should be noted that the height of the friction section orifice was gradually reduced from the second stage to the fourth stage, and the height reduction in each stage was determined based on experimental data.

The outlet diverging section in the fourth-stage flat streamlined friction–cavitation nozzle was configured based on the shear–cavitation–inception principle. The diverging angle is an important parameter affecting the cavitation performance of this nozzle. Previous studies have shown that shear cavitation nozzles can generally obtain relatively strong cavitation performance when the diverging angle is within the range of 45–60° [40]. Using the fourth-stage friction section orifice dimensions as the basis, the diverging angles of the outlet diverging section in the width and height directions were determined to be 49.4° and 60°, respectively. Both angles fall within the recommended optimal range, which is conducive to maintaining a relatively high cavitation intensity.

### 2.3. Numerical Method, Numerical Schemes, and Boundary Conditions

As the flake graphite slurry passes through the multi-stage friction–shear cavitating waterjet nozzle, shear cavitation is induced in the diverging section of the fourth-stage cavitation nozzle, producing a water–vapor–flake graphite three-phase cavitating waterjet. To compare the average flow characteristics of the three-phase flow field in the multi-stage friction–shear cavitating waterjet nozzle under different inlet pressures, the calculations were conducted with a pressure-based solver under steady-state conditions. Given the strong cavitation behavior, high vapor volume fraction, water–vapor–flake graphite three-phase flow, slurry volume concentration above 10%, and coexistence of liquid water, water vapor, and solid graphite particles in the flow field, the Eulerian multiphase model was selected to resolve the water–vapor–flake graphite three-phase flow [41]. Turbulence was modeled using the Realizable *k*–*ε* model, while the near-wall region was treated with the enhanced wall treatment [42]. The liquid–vapor phase change between the water phase and the water vapor phase was represented by the Schnerr–Sauer cavitation model. The numerical models, solution algorithm, and main discretization schemes used in this study are listed in Table 2.

As shown in Table 2, PRESTO! was used for the pressure term, QUICK for the volume fraction equation, and the second-order upwind scheme for the remaining governing equations. Pressure and velocity were coupled through the Phase Coupled SIMPLE algorithm [43]. The relaxation factors were slightly adjusted from their default values to ensure convergence. The residual convergence criterion was set to R ≤ 10^−6^, with the upper iteration limit specified as 1 × 10^4^ iterations. In addition, convergence was further assessed by monitoring the inlet–outlet mass flow imbalance and the stabilization of velocity, pressure, and vapor volume fraction at selected monitoring points.

The inlet and outlet boundaries of the multi-stage friction–shear cavitating waterjet nozzle were defined as pressure inlet and pressure outlet boundary conditions, respectively. The inlet pressures were set to 5, 15, 25, 35, and 45 MPa, and the inlet diameter was 5.3 mm. Based on the actual submerged depth in the experiment, the outlet gauge pressure was assigned as 5000 Pa, and the outlet diameter was set equal to the outlet diameter of the external flow domain, namely 20 mm. The turbulence parameters were specified using the hydraulic diameter and turbulence intensity. The inlet turbulence intensity and backflow turbulence intensity were both set to 5% based on empirical values. No-slip wall boundary conditions were imposed on the wall surfaces. Since a quarter-domain flow model was adopted, the two horizontal and vertical geometric symmetry planes were set as symmetry boundary conditions.

## 3. Mathematical Model

Within the nozzle outlet diverging section, cavitation induced by multi-stage friction–shear effects leads to the formation of a highly turbulent water–vapor–flake graphite three-phase cavitating waterjet. The Eulerian multiphase model was used to describe the coupling among different phases, where mass conservation for each phase is governed by the continuity equations, and interphase momentum-transfer terms are included in the momentum equations. The turbulent flow was represented by the Realizable *k*–*ε* model, and liquid–vapor mass transfer was calculated using the Schnerr–Sauer cavitation model.

### 3.1. Multiphase Flow Model

Based on the Eulerian multiphase model, the continuity and momentum equations are expressed as follows.

(1) Continuity equations for water vapor, water, and flake graphite [41,44].
  (4) $$\frac{\partial }{\partial t}\left(\alpha_{v}\rho_{v}\right)+\nabla \cdot \left(\alpha_{v}\rho_{v}v_{v}\right)=0$$
  (5) $$\frac{\partial }{\partial t}\left(\alpha_{w}\rho_{w}\right)+\nabla \cdot \left(\alpha_{w}\rho_{w}v_{w}\right)=0$$
  (6) $$\frac{\partial }{\partial t}\left(\alpha_{s}\rho_{s}\right)+\nabla \cdot \left(\alpha_{s}\rho_{s}v_{s}\right)=0$$
  where *t* is time (s); *α_v_*, *α_w_*, and *α_s_* are the volume fractions of the vapor phase, water phase, and flake graphite phase, respectively; *ρ_v_*, *ρ_w_*, and *ρ_s_* are the densities of the vapor phase, water phase, and flake graphite phase, respectively (kg/m^3^); and **v***_v_*, **v***_w_*, and **v***_s_* are the velocity vectors of the vapor phase, water phase, and flake graphite phase, respectively (m/s).
(2) Momentum equations for water vapor, water, and flake graphite [41,44].
  (7) $$\frac{\partial }{\partial t}\left(\alpha_{v}\rho_{v}v_{v}\right)+\nabla \cdot \left(\alpha_{v}\rho_{v}v_{v}v_{v}\right)=-\alpha_{v}\nabla p-\nabla \left(\alpha_{v}\tau_{v}\right)+\alpha_{v}\rho_{v}g+M_{i,v}$$
  (8) $$\frac{\partial }{\partial t}\left(\alpha_{w}\rho_{w}v_{w}\right)+\nabla \cdot \left(\alpha_{w}\rho_{w}v_{w}v_{w}\right)=-\alpha_{w}\nabla p-\nabla \left(\alpha_{w}\tau_{w}\right)+\alpha_{w}\rho_{w}g+M_{i,w}$$
  (9) $$\frac{\partial }{\partial t}\left(\alpha_{s}\rho_{s}v_{s}\right)+\nabla \cdot \left(\alpha_{s}\rho_{s}v_{s}v_{s}\right)=-\alpha_{s}\nabla p-\nabla \left(\alpha_{s}\tau_{s}\right)+\alpha_{s}\rho_{s}g+M_{i,s}$$
  where *p* is the pressure (Pa); *τ_w_*, *τ_v_*, and *τ_s_* are the shear stress tensors of the water phase, vapor phase, and flake graphite phase, respectively (Pa); and *M_i*,*v_*, *M_i*,*w_*, and *M_i*,*s_* are the interphase interaction force terms for the vapor phase, water phase, and flake graphite phase, respectively.

### 3.2. Turbulence Model

In the Realizable *k*–*ε* model, the governing transport equations for *k* and *ε* are expressed as follows.

(1) Turbulent kinetic energy *k* equation [45]:
  (10) $$\rho \frac{dk}{dt}=\frac{\partial }{\partial x_{i}}\left[\left(\mu +\frac{\mu_{t}}{\sigma_{k}}\right)\frac{\partial k}{\partial x_{i}}\right]+G_{k}+G_{b}-\rho \varepsilon -Y_{M}$$
  where *μ* is the dynamic viscosity (Pa·s); *μ_t_* is the turbulent viscosity (Pa·s); *σ_k_* is the turbulent Prandtl number for turbulent kinetic energy; *G_k_* is the generation of turbulent kinetic energy caused by the mean velocity gradients; *G_b_* is the generation of turbulent kinetic energy caused by buoyancy; and *Y_M_* represents the contribution of fluctuating dilatation in compressible turbulence to the overall dissipation rate.
(2) Dissipation rate *ε* equation [45]:
  (11) $$\rho \frac{d\varepsilon }{dt}=\frac{\partial }{\partial x_{i}}\left[\left(\mu +\frac{\mu_{t}}{\sigma_{\varepsilon }}\right)\frac{\partial \varepsilon }{\partial x_{i}}\right]+\rho C_{1}S\varepsilon -\rho C_{2}\frac{\varepsilon^{2}}{k+\sqrt{v\varepsilon }}+C_{1\varepsilon }\frac{\varepsilon }{k}C_{3\varepsilon }G_{b}$$
  (12) $$C_{1}=\max\left[0.43,\frac{\eta }{\eta +5}\right]$$
  (13) $$\eta =\frac{Sk}{\varepsilon }$$
  where *σ_ε_* is the turbulent Prandtl number for the dissipation rate; *S* is the modulus of the mean strain rate tensor, which characterizes the magnitude of the mean deformation rate of the fluid (s^−1^); *ν* is the kinematic viscosity (m^2^/s); and *C*_1*ε*_ and *C*_3*ε*_ are empirical constants. In the numerical simulations, *σ_k_* = 1.0, *σ_ε_* = 1.2, *C*_2_ = 1.9, *C*_1*ε*_ = 1.44 and *C*_3*ε*_ = 1.

### 3.3. Cavitation Model

The Schnerr–Sauer cavitation model used in this study is a liquid–vapor phase change mass transfer model based on Rayleigh–Plesset bubble dynamics. It can be coupled with the Eulerian multiphase model and is used to describe the evaporation and condensation processes between the water phase and the vapor phase.

(1) Vapor volume fraction transport equation [46]:
  (14) $$\frac{\partial }{\partial t}\left(\alpha_{v}\rho_{v}\right)+\nabla \cdot \left(\alpha_{v}\rho_{v}v_{v}\right)=R_{e}-R_{c}$$
  where *R_e_* is the evaporation rate, corresponding to cavitation inception and bubble growth (kg·m^−3^·s^−1^), and *R_c_* is the condensation rate, corresponding to bubble collapse (kg·m^−3^·s^−1^).
(2) Evaporation and condensation source terms [46]:
  (15) $$R_{e}=F_{vap}\frac{\rho_{w}\rho_{v}}{\rho }\alpha_{v}\alpha_{w}\frac{3}{R_{B}}\sqrt{\frac{2}{3}\frac{\left(P_{v}-P\right)}{\rho_{w}}},\;P_{v}\geq P$$
  (16) $$R_{c}=F_{cond}\frac{\rho_{w}\rho_{v}}{\rho }\alpha_{v}\alpha_{w}\frac{3}{R_{B}}\sqrt{\frac{2}{3}\frac{\left(P-P_{v}\right)}{\rho_{w}}},\;P_{v}\leq P$$
  where *F_vap_* and *F_cond_* are the empirical correction coefficients for evaporation and condensation rates, respectively, with default values of 1 and 0.2; *P* is the local far-field pressure (Pa); *P_v_* is the saturated vapor pressure (Pa); and *R_B_* is the general bubble radius (m).

## 4. Simulation Results and Discussion

### 4.1. Computational Domain and Mesh Generation

The full computational geometry was constructed in SolidWorks 2024 based on the internal flow channel geometry of the multi-stage friction–shear cavitating waterjet nozzle shown in Figure 1c. Because the nozzle and jet are symmetric about both the horizontal and vertical planes, and the analysis focuses on the flow field characteristics inside and outside the nozzle under different inlet pressures, a quarter-domain model was used for the numerical simulation. This treatment reduces the computational cost while preserving the main geometric features of the computational model. The external flow domain was specified with a length and diameter of 20 mm.

**Figure 3:**
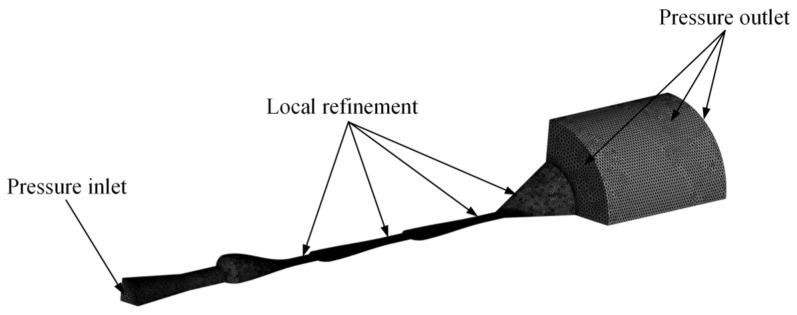
Computational mesh model.

The computational mesh was generated using ANSYS Meshing 2024 R2. Considering the flow domain geometry and the highly turbulent flow, the nozzle flow field was discretized using tetrahedral unstructured cells. Local mesh refinement was applied to the high-velocity regions, including the converging sections, friction sections, and the diverging section where strong vortical flow may occur. Figure 3 presents the computational mesh of the multi-stage friction–shear cavitating waterjet nozzle.

### 4.2. Grid Independence Test

In computational fluid dynamics simulations, mesh quality and resolution directly affect the accuracy and reliability of the numerical results. Insufficient mesh resolution may increase discretization errors and introduce or intensify numerical dissipation, thereby affecting the reliability of the simulation results. Meanwhile, excessive mesh refinement increases the computational cost. Therefore, a grid independence test is necessary. In this study, an inlet pressure of 25 MPa, which is the intermediate value among the five inlet pressure levels, was selected for the grid independence test. The maximum vapor volume fraction, maximum water phase axial velocity, and maximum flake graphite particle phase axial velocity were selected as the monitoring variables for the grid independence test. Six mesh schemes with different numbers of cells were used to examine the mesh sensitivity of the main monitoring indicators in the jet flow field. The equivalent full-domain cell counts and the corresponding numerical simulation results are listed in Table 3.

**Figure 4:**
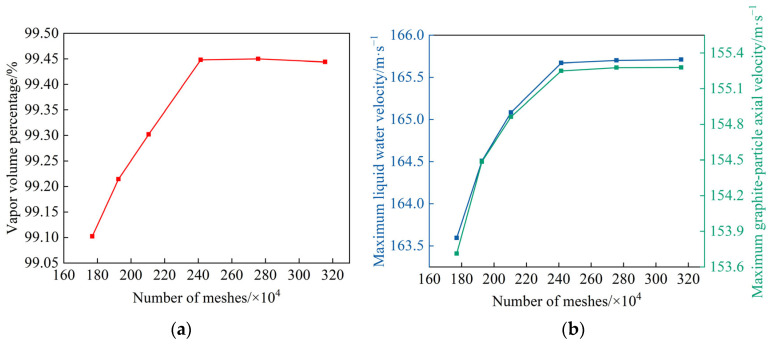
Variation curves of the three monitoring variables with the number of mesh cells. (**a**) Maximum vapor volume fraction; (**b**) maximum water phase axial velocity and maximum flake graphite particle phase axial velocity. In panel (**b**), the blue line represents the maximum water phase axial velocity, and the green line represents the maximum flake graphite particle phase axial velocity.

Figure 4 shows the variation curves of the maximum vapor volume fraction, maximum water phase axial velocity, and maximum flake graphite particle phase axial velocity with the number of equivalent full-domain mesh cells. As shown in Table 3 and Figure 4, when the number of full-domain mesh cells increased from 1,769,956 to 1,925,716, 2,105,540, and 2,414,900, the maximum vapor volume fraction and the two velocity monitoring variables were still affected by the mesh resolution, and relatively evident differences were observed. When the number of full-domain mesh cells exceeded 2,414,900, further mesh refinement caused a relative change of less than 0.1% in the maximum vapor volume fraction, maximum water phase axial velocity, and maximum flake graphite particle phase axial velocity. This indicates that the numerical results were essentially independent of mesh resolution and satisfied the grid convergence criterion. To balance computational accuracy and efficiency, the mesh scheme containing 2,414,900 equivalent full-domain cells and 475,908 mesh nodes was used in subsequent simulations.

### 4.3. Effect of Inlet Pressure on the Vapor Volume Fraction Field

**Figure 5:**
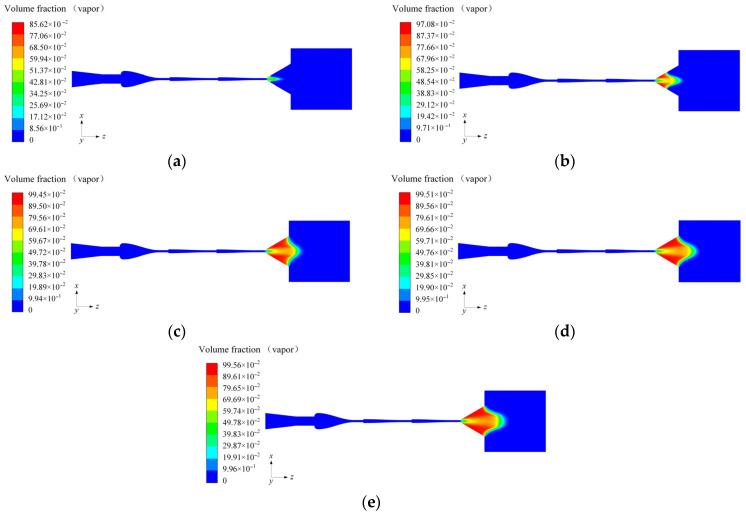
Vapor volume fraction contours under different inlet pressures. (**a**) 5 MPa; (**b**) 15 MPa; (**c**) 25 MPa; (**d**) 35 MPa; (**e**) 45 MPa.

Figure 5 illustrates the contours of vapor volume fraction in the jet flow field at nozzle inlet pressures of 5, 15, 25, 35, and 45 MPa. Under all tested inlet pressures, distinct cavitation was generated when the flake graphite slurry exited the multi-stage friction–shear cavitating waterjet nozzle. Cavitation inception occurred at the inlet of the diverging section in the friction–cavitation nozzle, where the local pressure initially fell below the saturated vapor pressure of water at the corresponding temperature, thereby forming cavitation bubbles. Subsequently, a stable cavitation zone developed along the wall of the diverging section, with the cavitation intensity increasing toward the wall. Similar near-wall cavitation behavior was reported by He et al. [38] and Gao et al. [39] for converging–diverging cavitation nozzles, in which jet cavitation was mainly concentrated near the wall of the nozzle diverging section. In addition, the near-wall region showed a much higher vapor volume fraction than the central region of the flow field, and a symmetric cavitation cloud appeared near the nozzle outlet. This distribution is consistent with the observation of Dong et al. [27], who studied the three-dimensional structure of an angular cavitation nozzle and reported that lower vapor volume fraction appeared in the central region, whereas higher values occurred in the surrounding region.

Mechanistically, this cavitation feature can be explained by the shear-induced cavitation mechanism generated by the high-speed jet. The high-pressure flake graphite slurry is progressively accelerated inside the nozzle and then enters the diverging section at high velocity. A sharp radial variation in the axial velocity is generated in the diverging section, resulting in the formation of a strong near-wall shear layer and turbulent vortex structures. In the local low-pressure region, incipient cavitation bubbles form in the jet and subsequently develop into a cavitating waterjet. When the jet flows out of the nozzle and enters the external flow field, its velocity gradually attenuates, while the local static pressure recovers along the axial direction. When the local pressure becomes higher than the saturated vapor pressure of water, the decrease in vapor volume fraction first appears near the region where the jet enters the external flow field, then gradually propagates toward the jet center, and eventually approaches zero. The downstream end of the vapor volume fraction contour exhibits an approximately bell-shaped distribution, longer in the central region and shorter on both sides.

The quantitative results in Figure 5 indicate that the maximum vapor volume fraction increased from 85.62% at 5 MPa to 97.08%, 99.45%, 99.51%, and 99.56% at 15, 25, 35, and 45 MPa, respectively. This indicates that the maximum vapor volume fraction increased with the nozzle inlet pressure. However, this pressure-induced increase was more pronounced at lower pressures and became weaker at higher pressures, suggesting that 5–25 MPa was an effective pressure range for improving the cavitation performance of the jet. Within this range, the maximum vapor volume fraction increased significantly, indicating that the cavitation intensity was markedly enhanced. When the inlet pressure exceeded 25 MPa, the maximum vapor volume fraction approached a saturation level and remained above 99%, indicating that cavitation had entered a relatively fully developed stage. At this stage, additional increases in inlet pressure contributed little to further increasing the vapor volume fraction. This trend agrees with the numerical results of Su et al. [37], who found that when the inlet pressure increased to 25 MPa, the vapor fraction reached 0.9924, and a further increase in pressure did not lead to a significant increase in vapor fraction. These results indicate that, at inlet pressures of 25 MPa and above, the nozzle can generate stable and distinct cavitation, suggesting a relatively strong cavitation-inducing capability of the multi-stage friction–shear cavitating waterjet nozzle designed in this study.

**Figure 6:**
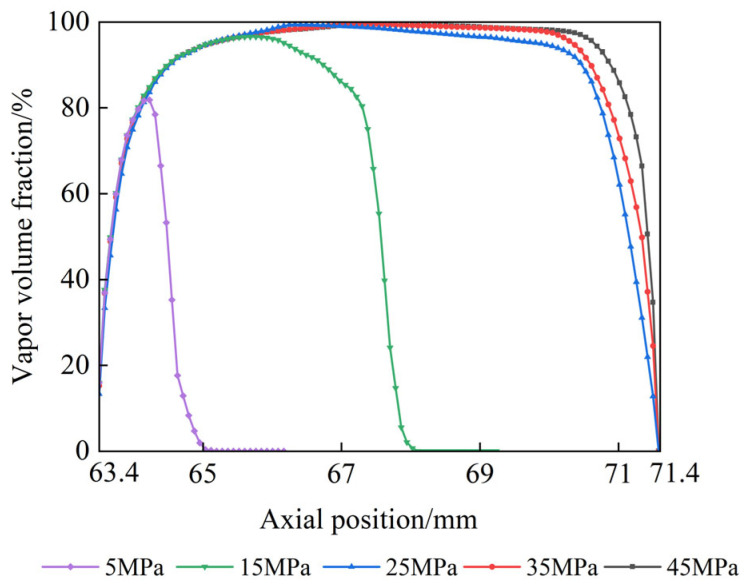
Variation in vapor volume fraction along the axial coordinate under different inlet pressures from the entrance to the outlet of the nozzle diverging section.

It should be noted that the multi-stage friction–shear cavitating waterjet nozzle designed in this study is intended for flake graphite liberation. Therefore, the size of the region with the maximum vapor volume fraction generated by the cavitating waterjet is also important for flake graphite liberation. Accordingly, the near-wall region with a high vapor volume fraction in the nozzle diverging section was further examined at different inlet pressures. Figure 6 presents the axial profiles of vapor volume fraction for the five inlet pressure conditions. As the inlet pressure increased, the near-wall high-vapor-volume-fraction region generated by jet cavitation expanded along the nozzle diverging section. This trend can be attributed to the higher jet velocity at the entrance of the diverging section under elevated inlet pressure. Under the same ambient pressure, a higher jet velocity results in slower velocity attenuation, a longer core region, and a longer region where shear interaction between the jet and the surrounding water induces turbulent vortex structures. Consequently, the cavitation-induced high-vapor-volume-fraction region expands over a larger range. This behavior agrees with Su et al. [37], who found that the vapor distribution region in the diverging section gradually expands along the wall of the diverging section. Previous studies by the authors demonstrated that the collapse of cavitation bubbles around flake graphite particles could generate microjets, stress waves, and local pressure fluctuations, thereby promoting interfacial separation between graphite flakes and gangue minerals [47]. Therefore, for the multi-stage friction–shear cavitating waterjet proposed in this study, the expansion of the high-vapor-volume-fraction region may increase the probability that cavitation bubble collapse acts on flake graphite particles and their interfaces, thereby providing favorable conditions for graphite–gangue interfacial liberation.

### 4.4. Effect of Inlet Pressure on the Axial Velocities of Water and Flake Graphite

For flake graphite liberation using the multi-stage friction–shear cavitating waterjet nozzle, the flake graphite slurry is driven by high-pressure water as pressure energy is converted into kinetic energy. Water phase axial velocity characterizes the jet cavitation intensity, whereas flake graphite particle phase axial velocity reflects the friction-assisted liberation behavior of flake graphite. Therefore, this section evaluates how inlet pressure changes the axial velocities of the water phase and flake graphite particle phase.

**Figure 7:**
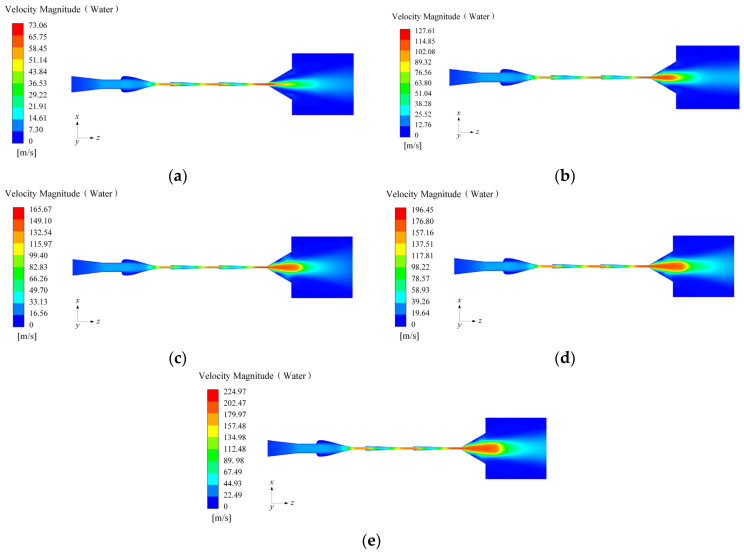
Axial velocity contours of the water phase under different inlet pressures. (**a**) 5 MPa; (**b**) 15 MPa; (**c**) 25 MPa; (**d**) 35 MPa; (**e**) 45 MPa.

Figure 7 presents the water phase axial velocity contours at nozzle inlet pressures of 5, 15, 25, 35, and 45 MPa. The contours show similar water phase velocity patterns under different inlet pressures, while the peak axial velocity in the nozzle flow field increases as the inlet pressure increases. Specifically, the water phase axial velocity inside the nozzle is jointly governed by operating conditions and fluid properties. The external factors mainly include the nozzle inlet pressure, outlet pressure, and nozzle geometry, whereas the internal factor is the physical properties of water. The multi-stage friction–shear cavitating waterjet nozzle developed in this study consists of four nozzle stages, and the differences in the geometry and dimensions of each stage determine the specific flow characteristics of water through the nozzle.

When water enters the first-stage nozzle from the outlet of the high-pressure pipe, the nozzle diameter decreases linearly in the flow direction within the conical converging section, thereby rapidly accelerating the water phase. In the cylindrical section, when the water flow enters from the conical converging section, an entrance region effect is generated due to inertia; however, the average axial velocity in the cylindrical section remains approximately constant. In the second- and third-stage friction nozzles as well as the fourth-stage friction–cavitation nozzle, the axial velocity of water undergoes alternating deceleration and acceleration as the flow passes through each stage because of the “diverging–converging” geometry from the inlet to the friction section. Meanwhile, as the height of the friction section decreases stepwise in the last three nozzle stages, the axial velocity gradually increases and reaches its maximum near the outlet of the friction section of the fourth-stage nozzle.

In the fourth-stage nozzle diverging section, water enters the surrounding quiescent water, thereby forming a submerged waterjet. Close to the nozzle outlet, the water phase axial velocity remains approximately equal to the outlet velocity. As the standoff distance increases, the momentum exchange within the water phase becomes more intense due to the viscous effect of water. The surrounding quiescent water is entrained into the jet, causing the jet width to gradually increase, while the jet axial velocity attenuates and eventually merges into the surrounding water. This trend agrees with the conclusion of Du et al. [23], who found that the fluid maintained the same outlet velocity within a certain distance after being discharged from the nozzle, and then the jet velocity decreased gradually due to the resistance of the surrounding water. At higher inlet pressures, the jet carries greater kinetic energy and shows stronger resistance to disturbance from the surrounding water. As a result, the jet width increases, the potential core region extends, and the velocity distribution in the high-velocity region becomes more uniform. Under the five inlet pressures, the corresponding maximum water phase axial velocities were 73.06, 127.61, 165.67, 196.45, and 224.97 m/s, respectively. A higher axial jet velocity corresponds to a lower cavitation number in the diverging section, which is more favorable for the formation of stable cavitation. Meanwhile, the enhancement of cavitation development may strengthen the local impact effects associated with bubble collapse, thereby providing favorable conditions for the liberation of flake graphite particles.

**Figure 8:**
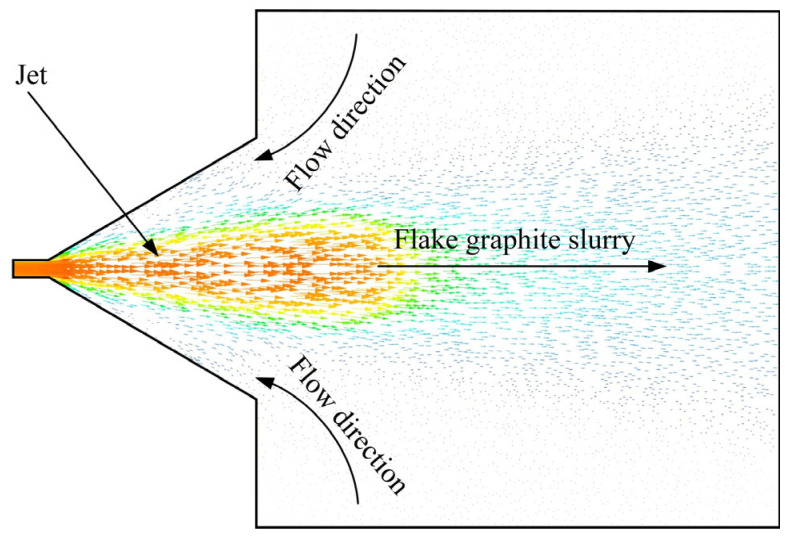
Velocity vector field of the water phase in the diverging section at an inlet pressure of 45 MPa. The color of the vectors qualitatively represents the velocity magnitude, with red indicating high-velocity regions and blue indicating low-velocity regions.

Figure 8 presents the water phase velocity vector field in the diverging section at a nozzle inlet pressure of 45 MPa. As shown in Figure 8, backflow of the surrounding quiescent water occurs adjacent to the wall of the nozzle diverging section and subsequently merges with the main flow toward the outlet of the diverging section. This phenomenon is mainly attributed to the pronounced velocity gradient at the submerged waterjet boundary in the nozzle diverging section, where intense distributed vortices are formed. The pressure at the vortex center is relatively low, forming a local low-pressure region, and the surrounding quiescent water flows toward this region under the pressure difference, resulting in backflow. In addition, the viscous effect of water also draws the surrounding quiescent water toward the jet center, further contributing to the formation of backflow. This observation agrees with Su et al. [37], who reported that a local backflow region forms in the nozzle diverging section when liquid flows out of the nozzle at a high velocity.

**Figure 9:**
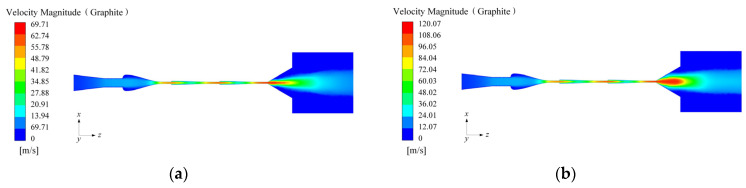

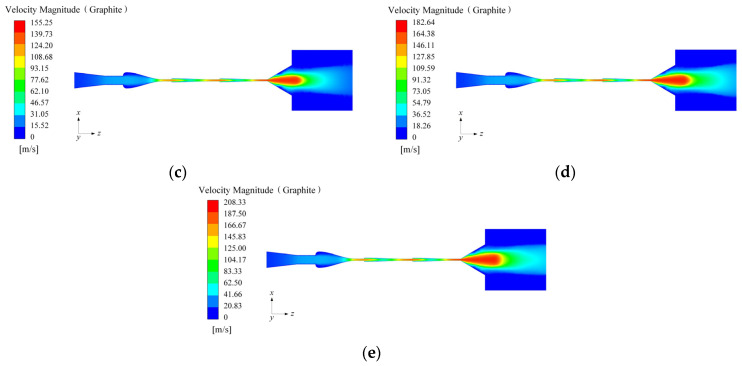
Axial velocity contours of the flake graphite phase under different inlet pressures. (**a**) 5 MPa; (**b**) 15 MPa; (**c**) 25 MPa; (**d**) 35 MPa; (**e**) 45 MPa.

Figure 9 presents the flake graphite particle phase axial velocity contours in the multi-stage friction–shear cavitating waterjet under different inlet pressures. The distributions show similar patterns at different inlet pressures, and the maximum axial velocity of the flake graphite particle phase increases as the inlet pressure increases. Comparison of Figure 7 and Figure 9 shows that, for the same inlet pressure and axial section, the flake graphite particle phase axial velocity distribution is generally consistent with the water phase distribution, and the main flow features remain similar. This behavior can be attributed to the liquid–solid two-phase hydraulic transport of the flake graphite slurry in the multi-stage friction–shear cavitating waterjet nozzle. Because the continuous water phase is pressurized and has a high axial velocity in the nozzle, while the flake graphite slurry has a volume concentration greater than 10% and contains fine graphite particles, the slurry flow can be treated, under the present model assumptions, as a relatively homogeneous transport process. In each nozzle stage, the acceleration and deceleration behavior of the flake graphite particles follows that of the water phase, and the particle velocity attains its maximum in the fourth-stage friction section. At inlet pressures of 5, 15, 25, 35, and 45 MPa, the corresponding maximum axial velocities of the flake graphite particles were 69.71, 120.07, 155.25, 182.64, and 208.33 m/s, respectively.

**Figure 10:**
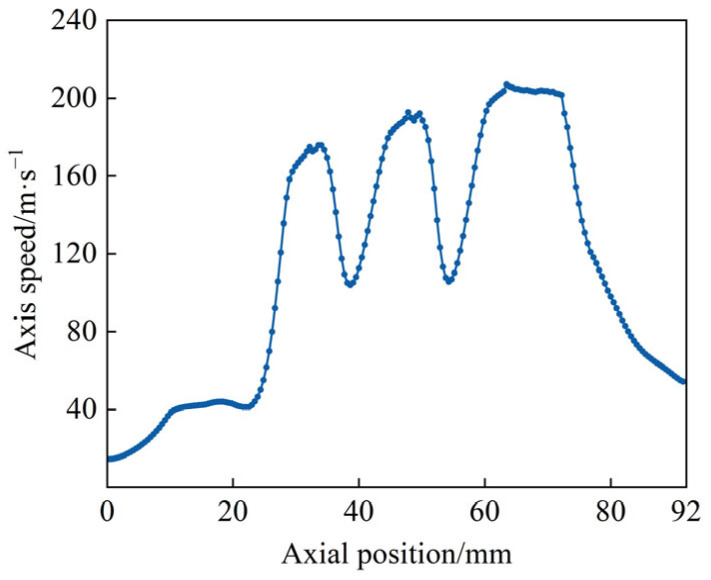
Variation in the centerline velocity of the flake graphite phase along the z-axis coordinate.

To further analyze the variation in the axial velocity of flake graphite particles within the computational domain, Figure 10 shows the centerline velocity of flake graphite particles as a function of the z-axis coordinate at a nozzle inlet pressure of 45 MPa. As shown in Figure 10, the centerline velocity of the flake graphite particles exhibits three distinct velocity peaks in the computational domain. Specifically, at the nozzle inlet, graphite particles enter the nozzle with a centerline velocity of 14.43 m/s under the action of the inlet pressure. In the conical converging section of the first-stage nozzle (*z* = 0–10 mm), the centerline velocity of graphite particles gradually increases under the drag force of water as the water velocity increases. In the cylindrical section (*z* = 10–17.5 mm), although the water velocity remains nearly constant, a velocity difference still exists between graphite particles and water. Therefore, the centerline velocity of graphite particles continues to increase under the drag force of water, but the rate of increase becomes significantly lower.

After graphite particles enter the second-stage nozzle, their centerline velocity decreases within the circular diverging section (*z* = 17.50–21.56 mm) owing to the increase in flow channel cross-sectional area. In the converging section, the reduced flow channel area causes the centerline velocity to increase. In the friction section, the centerline velocity continues to increase, but the rate of increase becomes lower. Moreover, because the centerline velocity of high-pressure water remains close to the outlet velocity in the initial jet region downstream of the friction section outlet, a velocity slip exists between graphite particles and water. As a result, the graphite particles continue to accelerate under the drag force of water and reach the first centerline velocity peak of 175.09 m/s near the outlet of the friction section at *z* = 32.19 mm.

Before the friction sections of the third- and fourth-stage nozzles, the cross-sections of the flow channels both show a “diverging–converging” variation. Therefore, the centerline velocity of graphite particles again undergoes a decrease followed by an increase in each stage, forming the second and third velocity peaks at *z* = 47.82 mm and *z* = 63.45 mm, respectively. At the third peak, the centerline velocity reaches 207.12 m/s, which is the maximum value along the curve. Subsequently, graphite particles enter the diverging section and the external flow field, where the centerline velocity gradually decreases under the combined effects of flow channel expansion in the diverging section and the surrounding static water pressure.

## 5. Experimental Evaluation of Flake Graphite Liberation Through Waterjet Treatment

**Figure 11:**
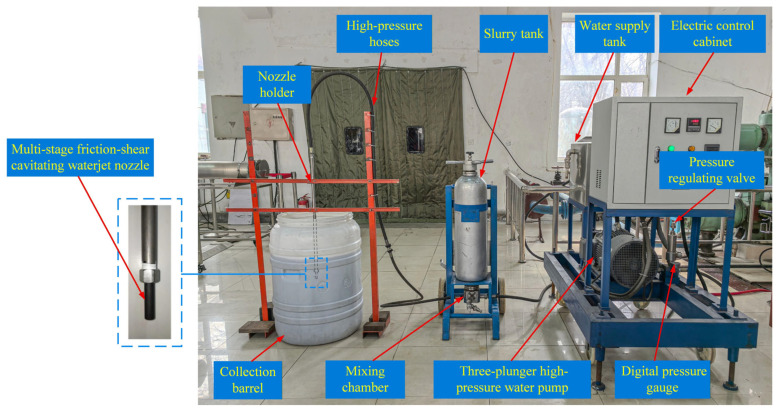
Experimental system for flake graphite liberation using the multi-stage friction–shear cavitating waterjet.

The numerical simulation results showed that the multi-stage friction–shear cavitating waterjet nozzle could form a distinct cavitation region in the outlet diverging section and enable the water phase and flake graphite particle phase to attain relatively high axial velocities inside the nozzle. These flow field characteristics indicate that the flow field distribution of the nozzle is favorable for enhancing the cavitation effect, fluid acceleration effect, and friction–shear action exerted on flake graphite particles inside the nozzle. To further evaluate the actual liberation effect of the nozzle on flake graphite, multi-stage friction–shear cavitating waterjet experiments for flake graphite liberation were carried out, and the experimental system is shown in Figure 11. The system mainly consists of an electric control cabinet, a water supply tank, a three-plunger high-pressure water pump (UDOR S.p.A., Rubiera, Italy), a digital pressure gauge, a pressure regulating valve, high-pressure hoses, a slurry tank, a mixing chamber, a nozzle holder, a multi-stage friction–shear cavitating waterjet nozzle, and a collection barrel. During the experiment, the electric control cabinet controlled the high-pressure water pump to draw and pressurize water. The water pressure was adjusted to the experimental pressure of 25 MPa using the pressure regulating valve and digital pressure gauge. The pressurized water then entered the bottom of the slurry tank through a high-pressure hose and mixed with the slurry discharged from the slurry tank in the mixing chamber. Subsequently, the mixed slurry passed through the high-pressure hose and nozzle holder, was ejected from the nozzle, and completed the liberation of flake graphite before finally entering the collection barrel.

Particle size distribution and fixed carbon content were selected as evaluation indicators to evaluate the effect of the proposed nozzle on the separation of flake graphite from gangue minerals. The sample was obtained from the first-stage cleaning product of the Jixi Liumao graphite concentrator in China. The flake graphite particles were surface-cleaned with anhydrous ethanol to remove residual flotation reagents from the particle surfaces, thereby reducing the influence of residual reagents on particle wettability, dispersion state, and the jet liberation process. The prepared sample was then used for the subsequent experiments. The jet-liberated product was floated using an XFD-II single-cell flotation machine (Changsha Shunze Mining and Metallurgical Machinery Manufacturing Co., Ltd., Changsha, China) to obtain the flake graphite flotation concentrate. The detailed flotation procedure was as follows. First, the flake graphite slurry after waterjet treatment was subjected to solid–liquid separation using a vacuum filter to remove excess water. The obtained solid sample was then dried to constant weight in an electrothermal constant temperature blast drying oven and used as the sample for the subsequent flotation tests. For each flotation test, 30 g of the dried sample was weighed and added to the 1.5 L flotation cell of the XFD-II single-cell flotation machine, and water was added to the specified volume to obtain a slurry concentration of 20 g/L. Kerosene was used as the collector and sec-octyl alcohol was used as the frother, with dosages of 0.05 mL and 11 μL, respectively. Before flotation, the impeller speed of the flotation machine was adjusted to 1600 r/min, and the aeration rate was set to 200 L/h. With the aeration valve closed, the flake graphite sample and water were first fully mixed. Kerosene was then added and conditioned for 2 min, followed by the addition of sec-octyl alcohol and conditioning for 30 s. Subsequently, the aeration valve was opened and the scraper was started for flotation. The froth scraping time was 3 min, and the collected froth product was used as the flake graphite flotation concentrate.

The size characteristics of the flake graphite particles were evaluated through particle size distribution analysis. A 5 g sample was taken from both the feed sample and the waterjet-treated flotation concentrate, according to GB/T 3520-2008 [48], and analyzed using a L1064 laser particle size analyzer (Cilas, Orléans, France). The particle size distribution results are listed in Table 4. As shown in Table 4, the D10, D50, and D90 particle sizes of the feed sample were 15.64, 51.35, and 121.36 μm, respectively, whereas those of the waterjet-treated flotation concentrate were 14.41, 49.14, and 103.33 μm, respectively. Compared with the feed sample, the D10, D50, and D90 particle sizes of the waterjet-treated flotation concentrate decreased by 1.23, 2.21, and 18.03 μm, respectively, and the average particle size decreased by 7.76 μm.

**Figure 12:**
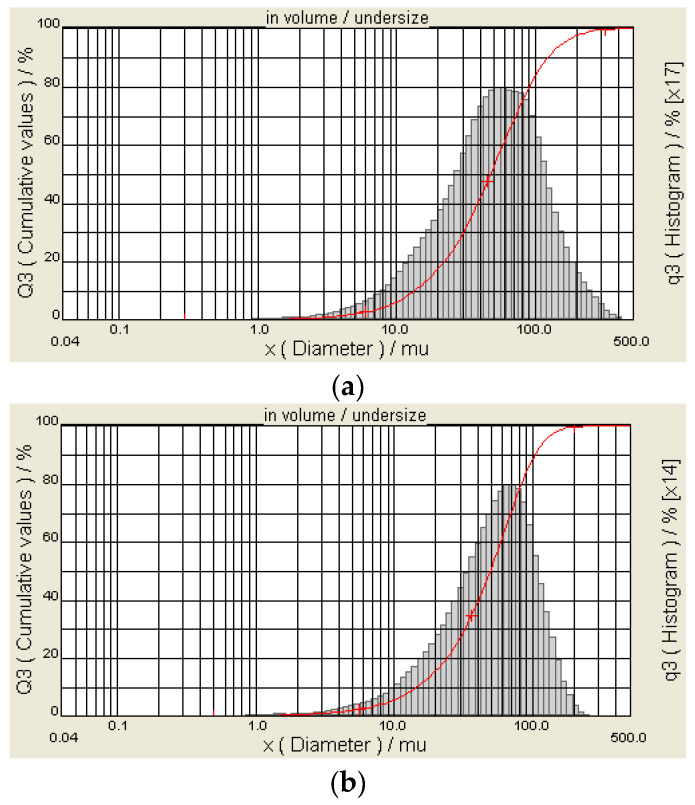
Cumulative particle size distribution curves of the feed sample and the waterjet-treated flotation concentrate. (**a**) Cumulative particle size distribution curve of the feed sample; (**b**) cumulative particle size distribution curve of the waterjet-treated flotation concentrate. The red line with “+” symbols represents the cumulative particle size distribution, and the gray bars represent the particle size histogram.

Figure 12 presents the cumulative particle-size distribution curves for flake graphite particles from the feed sample and the waterjet-treated flotation concentrate. As shown in Figure 12, compared with the feed sample, the waterjet-treated flotation concentrate exhibited a narrower particle size distribution, mainly concentrated within the range of 40–100 μm. When the particle size was approximately 80 μm, the cumulative volume distribution approached 80%, indicating a more concentrated overall particle size distribution. In addition, the fraction of particles larger than 100 μm was clearly reduced. These particle size results indicate that the waterjet-treated flotation concentrate exhibited a finer and more concentrated particle size distribution.

The fixed carbon content was used as an indicator to evaluate the quality of flake graphite [49]. To improve the accuracy of fixed carbon content determination, the feed sample and flotation concentrates after waterjet treatment were each divided into two groups. A 1 g sample was taken from each group for fixed carbon content analysis. The average value of the two measured fixed carbon contents was used as the fixed carbon content of the corresponding feed sample or waterjet-treated flotation concentrate, and the standard deviation of the two measurements was used to characterize the measurement uncertainty. The fixed carbon content results are summarized in Table 5. Table 5 shows that the fixed carbon content of the feed sample was 49.11 ± 0.43%, whereas that of the waterjet-treated flotation concentrate was 78.77 ± 0.05%. The corresponding relative standard deviations were 0.88% and 0.06%, respectively, indicating good repeatability of the fixed carbon content measurements. The average fixed carbon content of the waterjet-treated flotation concentrate was 29.66 percentage points higher than that of the feed sample, corresponding to a relative increase of 60.40%. This indicates that the self-developed multi-stage friction–shear cavitating waterjet nozzle can achieve surface liberation between flake graphite and gangue minerals, effectively remove gangue impurities embedded on the graphite surface, and thereby substantially improve the concentrate grade.

**Figure 13:**
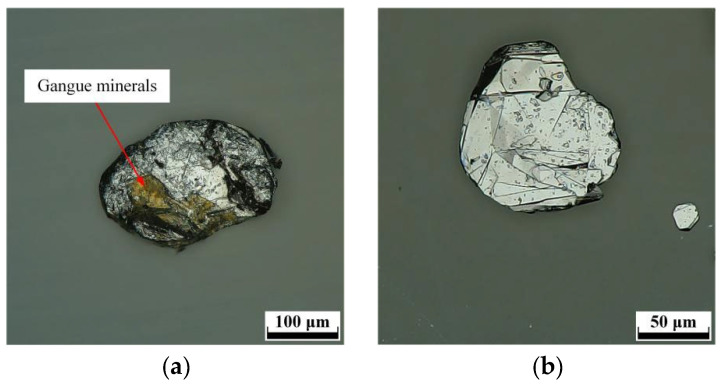
Comparison of the microscopic morphology of flake graphite particle surface states in the feed sample and in the waterjet-treated flotation concentrate. (**a**) Flake graphite particles in the feed sample, with gangue minerals attached to the surface; (**b**) flake graphite particles in the concentrate after waterjet treatment and flotation, with markedly reduced gangue attachment on the surface.

To further visually demonstrate the effect of the multi-stage friction–shear cavitating waterjet on flake graphite liberation, Figure 13 presents a microscopic morphology comparison of the surface states of flake graphite particles in the feed sample and in the concentrate after waterjet treatment and flotation. As shown in Figure 13a, gangue minerals are clearly attached to the surfaces of some flake graphite particles in the feed sample. Figure 13b shows that the surfaces of flake graphite particles in the concentrate after waterjet treatment and flotation are relatively clean, with markedly reduced gangue attachment. This microscopic morphological change is consistent with the fixed carbon content results, further indicating that the multi-stage friction–shear cavitating waterjet is beneficial for promoting the liberation of flake graphite from gangue minerals.

## 6. Conclusions and Future Directions

To improve the liberation degree between flake graphite and gangue minerals, a multi-stage friction–shear cavitating waterjet nozzle was designed and fabricated in this study. Computational fluid dynamics simulations were conducted to solve the water–vapor–flake graphite three-phase flow field of the nozzle and to analyze the cavitation distribution, water phase flow, and flake graphite particle phase motion characteristics inside the nozzle. Meanwhile, flake graphite liberation experiments were carried out to evaluate the actual liberation effect of the self-developed multi-stage friction–shear cavitating waterjet nozzle. The main conclusions are as follows:

(1) Distinct cavitation developed in the diverging section of the multi-stage friction–shear cavitating waterjet nozzle. The cavitation intensity increased toward the wall of the diverging section, and the vapor volume fraction in the near-wall region was markedly higher than that in the central flow region. When the inlet pressure increased from 5 to 45 MPa, the high-vapor-volume-fraction region gradually expanded, and the maximum vapor volume fraction increased from 85.62% to 99.56%. At inlet pressures of 25 MPa and above, the maximum vapor volume fraction remained above 99%. These results indicate that the self-developed multi-stage friction–shear cavitating waterjet nozzle has a relatively strong cavitation-inducing capability.
(2) Increasing the inlet pressure significantly increased the water phase axial velocity. At inlet pressures of 5, 15, 25, 35, and 45 MPa, the corresponding maximum water phase axial velocities were 73.06, 127.61, 165.67, 196.45, and 224.97 m/s, respectively. The water phase underwent alternating deceleration and acceleration inside the multi-stage nozzle under the influence of the “diverging–converging” structures and reached a relatively high velocity near the outlet of the friction section of the fourth-stage nozzle. This provided favorable flow conditions for cavitation development in the diverging section and particle acceleration.
(3) The axial velocity distributions of the flake graphite particle phase under different inlet pressures showed similar patterns. At inlet pressures of 5, 15, 25, 35, and 45 MPa, the corresponding maximum axial velocities of the flake graphite particle phase were 69.71, 120.07, 155.25, 182.64, and 208.33 m/s, respectively. At an inlet pressure of 45 MPa, the centerline velocity of the flake graphite particles exhibited three distinct peaks along the nozzle axis and reached the maximum value of 207.12 m/s at the third peak. This indicates that the multi-stage contraction, friction, and expansion structures enabled the flake graphite particles to undergo multiple acceleration processes inside the nozzle, thereby helping to enhance the friction–shear action between particles and the flow channel wall as well as among particles.
(4) Multi-stage friction–shear cavitating waterjet treatment promoted the liberation of flake graphite from gangue minerals. At an inlet pressure of 25 MPa, after waterjet treatment and flotation, the fixed carbon content of the flake graphite concentrate increased from 49.11% in the feed sample to 78.77%, corresponding to an increase of 29.66 percentage points. The D90 particle size decreased from 121.36 to 103.33 μm, and the average particle size decreased from 62.78 to 55.02 μm. These results indicate that the self-developed multi-stage friction–shear cavitating waterjet nozzle can effectively promote the liberation of flake graphite from gangue minerals, thereby removing gangue impurities embedded in graphite and improving the concentrate grade.

Based on the above results, future studies may further combine transient numerical simulation, high-speed photography, particle image velocimetry (PIV), and pressure measurement to experimentally measure the dynamic evolution of cavitation clouds, velocity field distribution, and pressure pulsation characteristics at the nozzle outlet and in the near-wall region, thereby obtaining quantitative data that can be directly compared with CFD simulation results. On this basis, statistical metrics such as RMSE and MAE may be further used to quantitatively evaluate the differences between the simulated cavitation-related characteristic parameters, such as velocity, pressure, or vapor volume fraction, and the experimental measurements, thereby improving the quantitative reliability of numerical model validation. In addition, future work may further optimize the structures of the friction sections at different stages, the outlet diverging section, the inlet pressure, and the slurry concentration, using fixed carbon content, particle size composition, large flake retention rate, and energy consumption as comprehensive evaluation indicators. These studies would provide a basis for the engineering application and process optimization of the multi-stage friction–shear cavitating waterjet nozzle.

## Figures and Tables

**Table 1:** Physical parameters of each phase in the Eulerian multiphase model.

Phase Name	Phase Definition	Density (kg·m^−3^)	Dynamic Viscosity (Pa·s)	Volume Fraction	Representative Particle Diameter (mm)
Liquid water	Primary phase	1000	1 × 10^−3^	Initial inlet volume fraction: 0.85	Not applicable
Water vapor	Gaseous secondary phase	0.02558	1 × 10^−5^	Initial inlet volume fraction: 0; generated by the cavitation phase change process	Not applicable
Flake graphite particles	Solid secondary phase	2100	Not applicable; particle phase transport is described by the Eulerian solid phase model	Initial inlet volume fraction: 0.15	0.062

**Table 2:** Numerical models, solution algorithms, and discretization schemes.

Setting Item	Specific Setting
Solver type	Pressure-based solver
Time treatment	Steady-state solution
Multiphase flow model	Eulerian multiphase model
Turbulence model	Realizable *k*–*ε* model
Near-wall treatment	Enhanced wall treatment
Cavitation model	Schnerr–Sauer model
Pressure–velocity coupling method	Phase Coupled SIMPLE
Pressure discretization scheme	PRESTO! scheme
Volume fraction discretization scheme	QUICK scheme
Momentum equation discretization scheme	Second-order upwind scheme
Turbulent kinetic energy equation discretization scheme	Second-order upwind scheme
Turbulent dissipation rate equation discretization scheme	Second-order upwind scheme

**Table 3:** Grid independence test results.

Number of Equivalent Full-Domain Mesh Cells	Maximum Vapor Volume Fraction (%)	Maximum Water Phase Axial Velocity (m·s^−1^)	Maximum Flake Graphite Particle Phase Axial Velocity (m·s^−1^)
1,769,956	99.10	163.59	153.71
1,925,716	99.21	164.51	154.49
2,105,540	99.30	165.08	154.86
2,414,900	99.45	165.67	155.25
2,756,492	99.45	165.70	155.28
3,155,588	99.44	165.71	155.28

**Table 4:** Particle size distribution analysis results.

Characteristic Particle Size	Feed Sample (μm)	Waterjet-Treated Flotation Concentrate (μm)
D10	15.64	14.41
D50	51.35	49.14
D90	121.36	103.33
Average	62.78	55.02

**Table 5:** Fixed carbon content determination results and measurement uncertainties.

Flake Graphite Sample	Fixed Carbon Content in the First Determination (%)	Fixed Carbon Content in the Second Determination (%)	Average Fixed Carbon Content ± Standard Deviation (%)	Relative Standard Deviation (%)
Feed sample	48.81	49.42	49.11 ± 0.43	0.88
Waterjet-treated flotation concentrate	78.81	78.74	78.77 ± 0.05	0.06

## Data Availability

The data presented in this study are available on request from the corresponding author. As the data in this paper belongs to the National Natural Science Foundation of China, it involves related privacy and is not owned by individuals.
